# How the zebrafish got its stripes

**DOI:** 10.7554/eLife.14239

**Published:** 2016-02-15

**Authors:** Kelly A McGowan, Gregory S Barsh

**Affiliations:** 1 HudsonAlpha Institute for Biotechnology, Huntsville, United States; 1 HudsonAlpha Institute for Biotechnology, Huntsville, United Statesgbarsh@hudsonalpha.org; 2 Department of Genetics, Stanford University School of Medicine, Stanford, United States; 2 Department of Genetics, Stanford University School of Medicine, Stanford, United States

**Keywords:** pigment pattern, melanophore, xanthophore, evolution, danio, Zebrafish

## Abstract

Live-cell imaging and genetic tools reveal a new way in which pigment cells communicate in zebrafish

**Related research article** Eom DS, Bain EJ, Patterson LB, Grout ME, Parichy DM. 2015. Long-distance communication by specialized cellular projections during pigment pattern development and evolution. *eLife ***4**:e12401. doi: 10.7554/eLife.12401

**Image** Three types of pigment cells in the skin of zebrafish arrange themselves into a striped pattern

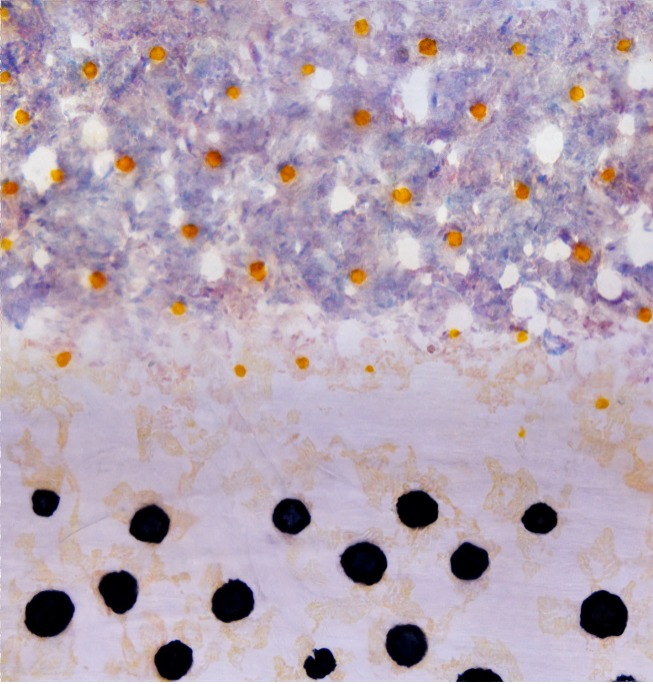

How the zebra got its stripes is a frequent topic in popular literature about the natural world. For developmental biologists, however, it is much easier to study the biology of pattern formation in zebrafish, where the tools of experimental embryology and genetics have proved to be very useful. Now, in eLife, David Parichy from the University of Washington and co-workers – including Dae Seok Eom as first author – report an important step forward in our understanding of pattern formation (Eom et al., 2015). Making use of recent advances in imaging technology and sophisticated genetic tools they reveal that a previously unappreciated form of communication between cells plays a critical role in the formation of stripes in zebrafish.

The elegant stripe patterns seen in adult zebrafish start to emerge in the juvenile fish, when the cells responsible for the different colors organize themselves into intricate patterns. Pigment cells called melanophores form the dark stripes, while xanthophores and iridophores form the regions between stripes. How does this come about? Work from several groups over the last decade has highlighted three general principles. First, although all the pigment cells originate in the same area of the zebrafish embryo, they have unique and separate developmental histories. For example, the melanophores and iridophores in adult fish belong to lineages that are separate from the pigment cells found in larvae (Mahalwar et al., 2014; McMenamin et al., 2014).

Second, genetic mutations that act primarily on one type of pigment cell can alter the distribution of other types of pigment cells in the adult fish (Parichy and Turner, 2003; Nakamasu et al., 2009; Patterson and Parichy, 2013; Frohnhofer et al., 2013). This demonstrates that a network of interactions is required for the proper stripe pattern to form. Finally, several zebrafish mutants that have different pigment patterns – for example, broad irregular stripes or spots instead of stripes – have defects in molecules that allow cells to communicate or attach to each other (Watanabe et al., 2006; Eom et al., 2012). This suggests that direct interactions between cells are a critical component of pattern formation.

Eom et al.’s findings add an intriguing new wrinkle to the story. They used time-lapse imaging to follow genetically marked melanophores, xanthophores and iridophores in the skin of young zebrafish as the adult stripes begin to form. The experiments used a new type of instrument – a spinning disk confocal laser microscope – that allows images to be acquired more rapidly than with the conventional scanning approach, and is especially powerful when coupled with the genetic tools available in zebrafish.

When Eom et al. looked at zebrafish that had been genetically engineered to express a fluorescent marker specifically in xanthophore cell membranes, they noticed thin filaments projecting from the xanthophores. These filaments sometimes extended for hundreds of microns, seemingly to deliver small packets of cargo (vesicles) to melanophores. Eom et al. found that these filaments, which contain cytoplasm, are distinct from previously known projections and dubbed them 'airinemes'. (This name pays tribute to the astronomer Sir George Biddel Airy, the Greek goddess Iris and the Iliad!)

A key aspect of pattern development in zebrafish is that the cells that will become the melanophores and xanthophores – which are initially disorganized in the skin – move in a carefully directed manner to form the adult stripes. Eom et al. realized that the majority of airinemes were produced by immature xanthophores, which then contacted immature melanophores located in between stripes. By using a genetic trick to prevent the production of airinemes, Eom et al. demonstrated that these structures play critical roles in the formation of clearly defined stripes. When the airnemes are missing, the immature melanophores persist in the regions between the stripes, and the edges of the stripes are ragged and ill-defined (Figure 1). A second genetic trick – manipulating a cell communication system called Notch signaling in melanophores – led the researchers to suggest that the interaction between xanthophores and melanophores involves a Notch signaling system.

**Figure 1.**
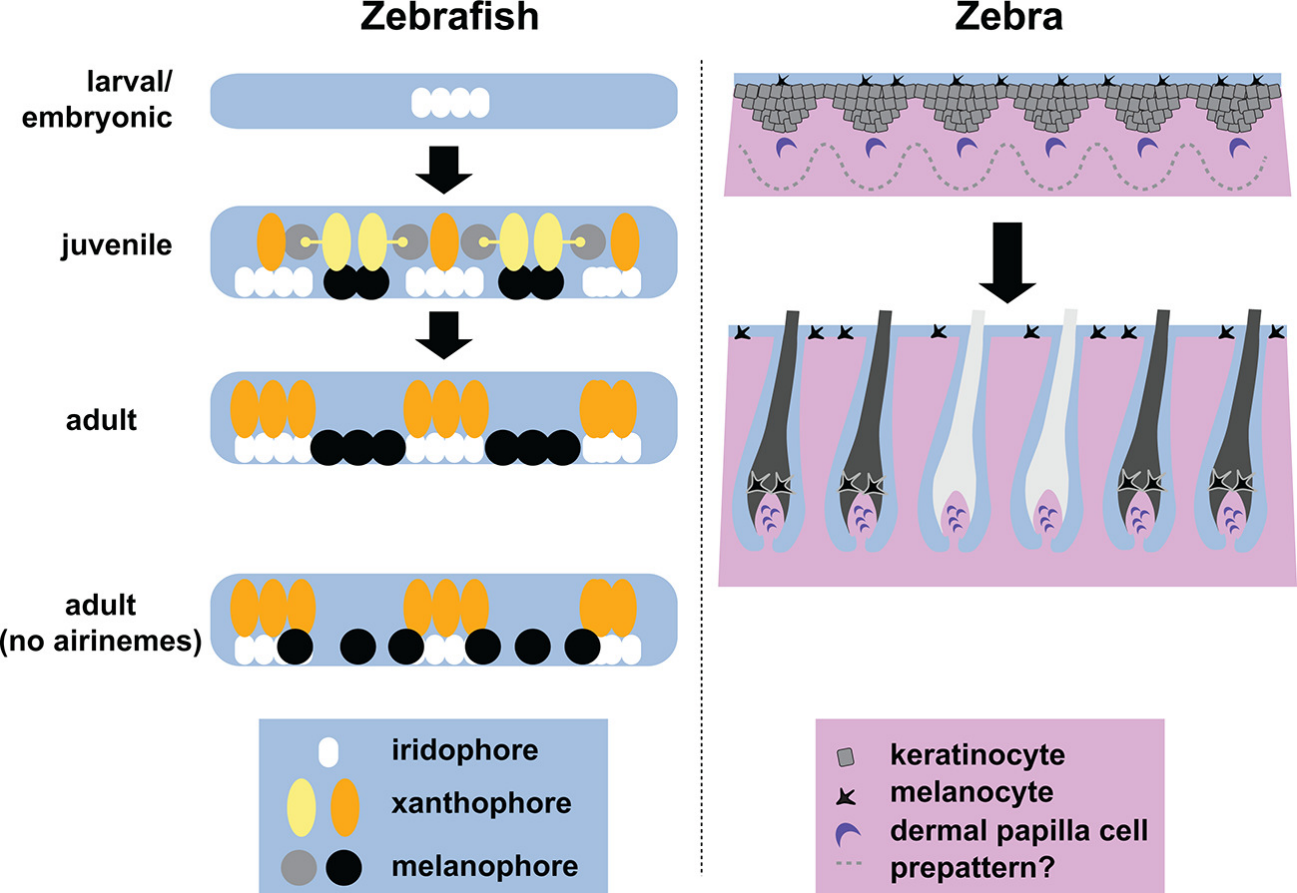
Stripe formation in zebrafish and zebra. Dense iridophores (white) cluster in the skin of zebrafish larvae (top left). In juvenile zebrafish, the different pigment cells begin to form stripes, during which immature xanthophores (yellow) extend airinemes to immature melanophores (gray). In adult zebrafish, mature xanthophores (orange) and iridophores form the pale interstripe regions, while mature melanophores (black) form the dark stripe regions. If airinemes are absent, some of the melanophores remain in the interstripe regions (bottom left, Eom et al., 2015). In zebras, epidermal cells (gray) and melanocytes (black) are uniformly distributed, and assignment of stripe identity may depend on periodic changes in the concentration of a Turing-like molecule outside the cells (dotted line) as the skin develops in the fetus. In adult zebra skin, work in horses and other equine animals (Imsland et al., 2015) suggests that hair follicle melanocytes are lost from white stripes.

While the genetic tools employed by Eom et al. are incredibly sophisticated, we are most excited about how they have been applied in the context of live-cell imaging to reveal a new form of cellular communication. This underscores one of the most important principles in biology—to look carefully—and raises the obvious question as to where should one look next?

An unsolved mystery of color pattern biology is how the appearance of stripes or spots can be remarkably stable within a species, yet evolutionarily flexible between species. For example, all leopards have rosette markings, but the same underlying mechanism can give rise to stripes in tigers or spots in cheetahs. One possible explanation stems from Alan Turing's work on chemical reaction-diffusion systems in the early 1950s: Turing showed how stable patterns can develop spontaneously in chemical systems, and that small changes in the components of the system can lead to large changes in the patterns produced.

A role for this type of system in zebrafish is somewhat controversial, in part because it doesn’t account for the different types of pigment cells involved. However, the same may not hold true for zebras and other mammals in which a single type of pigment cell is uniformly distributed in the skin.

Zebras will never enjoy the advantages of zebrafish when it comes to experimental genetics and embryology. However, advances in DNA sequencing are furthering the reach of observational genetics, perhaps even to exotic mammals with diverse color patterns like civets and servals. The question "How the zebra got its stripes" may not have the same answer as the question "How the zebrafish got its stripes". Vive la différence.
